# One Year after the Flood: Prevalence and Correlates of Post-Traumatic Stress Disorder among Residents in Fort McMurray

**DOI:** 10.3390/bs12030069

**Published:** 2022-03-02

**Authors:** Wanying Mao, Ejemai Eboreime, Reham Shalaby, Nnamdi Nkire, Belinda Agyapong, Hannah Pazderka, Gloria Obuobi-Donkor, Medard Adu, Ernest Owusu, Folajinmi Oluwasina, Yanbo Zhang, Vincent I. O. Agyapong

**Affiliations:** 1 Department of Psychiatry, University of Alberta, Edmonton, AB T6G 2B7, Canada; wmao2@ualberta.ca (W.M.); eboreime@ualberta.ca (E.E.); rshalaby@ualberta.ca (R.S.); nnamdi.nkire@albertahealthservices.ca (N.N.); bagyapon@ualberta.ca (B.A.); hannah@ualberta.ca (H.P.); obuobido@ualberta.ca (G.O.-D.); medard@ualberta.ca (M.A.); eowusu2@ualberta.ca (E.O.); folajinm@ualberta.ca (F.O.); yanbo9@ualberta.ca (Y.Z.); 2 Global Psychological E-Health Foundation, Edmonton, AB T6G 2B7, Canada; 3 Department of Psychiatry, Faculty of Medicine, Dalhousie University, 5909 Veterans Memorial Lane, 8th Floor Abbie J. Lane Memorial Building, QEII Health Sciences Centre, Halifax, NS B3H 2E2, Canada

**Keywords:** PTSD, trauma, flood, natural disaster, mental health, support, Fort McMurray

## Abstract

Background: The 2020 Fort McMurray (FMM) and area flood caused more than $228 million in insured damage, affected over 1200 structures, and more than 13,000 people were evacuated. Objective: This study sought to determine the prevalence of post-traumatic stress disorder (PTSD)-like symptoms and the risk predictors among the population of FMM one year after the 2020 flooding. Methods: An online quantitative cross-sectional survey was distributed to residents of FMM via REDCap between 24 April to 2 June 2021 to collect sociodemographic, clinical, and flood-related information. The PTSD checklist for DSM-5 (PCL-C) was used to assess likely PTSD among respondents. Results: 186 of 249 respondents completed all essential self-assessment questionnaires in the analysis, yielding a response rate of 74.7%. The prevalence of likely PTSD was 39.6% (65). Respondents with a history of depression were more likely to develop PTSD symptoms (OR = 5.71; 95% CI: 1.68–19.36). Similarly, responders with limited and no family support after the disaster were more prone to report PTSD symptoms ((OR = 2.87; 95% CI: 1.02–8.05) and (OR = 2.87; 95% CI: 1.06–7.74), respectively). Conclusions: Our research indicated that history of depression and the need for mental health counseling significantly increased the risk of developing PTSD symptoms following flooding; family support is protective. Further studies are needed to explore the relations between the need to receive counseling and presenting with likely PTSD symptoms.

## 1. Introduction

Natural disasters have impacted many people worldwide throughout history and have always caused peaks in mortality and morbidity [1]. Since 1990, natural disasters have affected around 217 million people each year [2]; approximately 300 million people live with violent insecurity worldwide [1]. More than 43% of Canadians experienced a major disaster in their lifetime, 3% reported being exposed to wildfire, and 1% had experienced major residential fires [2]. Over recent decades, natural disasters have occurred more frequently, and the scale has expanded more than ever before [1]. The reasons might boil down to increased urbanization rates, deforestation, environmental degradation, intensifying climate variables, such as higher temperatures, extreme precipitation, and more violent wind and water storms [1,3].

### 1.1. Flooding and Mental Health Issues

Floods account for approximately forty percent of natural disasters [4]. They are the most widespread weather-related natural disasters in developed and developing countries. Usually, they occur when an overflow of water submerges typically dry land [5]. Floods can happen following heavy rains, when huge ocean waves come on the shore, when the snow melts quickly, or when dams or levees collapse, and can last within minutes or over a long period (NSSL). The devastating impact of floods is evident in the floods in China (1959), Bangladesh (1974) and the tsunami in Southeast Asia in December 2004 [6]. In 2011, the Health Protection Agency (HPA) reviewed 48 research papers and those relevant government and non-government guidance documents published between 2004 and 2010, intended to assess and appraise the epidemiological evidence on flooding and mental health. The study indicated that flooding affects people of all ages, can exacerbate, or provoke mental health problems such as PTSD, depression, anxiety, panic disorder, etc., and highlights the importance of secondary stressors in prolonging the psychosocial impacts of flooding [7].

In the last two decades, studies have shown that the immediate impact of flood-related property damages, casualty and physical injuries may result in lasting mental health problems, including posttraumatic stress disorder (PTSD), depression, anxiety, and substance use disorders [3,8,9,10,11,12,13]. PTSD is among the most common post-disaster psychopathologies [3,14]. Research has documented the association between flooding and increased incidents of mental health illness, especially PTSD among survivors. A meta-analysis showed that among 40,600 flood victims, 3862 were diagnosed with PTSD after flooding; the combined incidence of PTSD was 15.74% [15]. PTSD symptoms and anxiety may last many years after the flooding [16,17]. A variety of risk factors increase the prevalence of PTSD symptoms in the aftermath of flooding, including socioeconomic factors [18], individual characteristics [19], mental health problem history [20], preparedness [21] and the severity of disaster [18,22].

### 1.2. Disaster Events and People in Fort McMurray

Fort McMurray (FMM) is in the Regional Municipality of Wood Buffalo (RMWB) in Northern Alberta, Canada, with a diverse population of 111,687 in 2018 [23], mainly in temporary project accommodations and in adjoining rural communities [24]. On 26 April 2020, the RMWB declared a state of local emergency (SOLE) after flooding the northern Alberta city’s downtown and surrounding areas, caused by a 25-kilometre-long ice jam formed in the Athabasca River [25]. This flood caused over $228 million insured damages, destroyed 1200 structures and forced 13,000 residents to leave their homes [25]. At the same time, FMM was heavily affected by the COVID-19 pandemic [26]. The COVID-19 pandemic may profoundly affect an individual’s physical and mental health [27,28]. Physical distancing and sanitation measures of COVID-19 also posted further challenges on evacuation, post-disaster regulation and recovery [29]. These physical and economic costs of such traumatic experiences always accompany emotional difficulties and stress [30] and dampen survivors’ mental health and general well-being [27,30,31]. One example is FMM’s wildfires associated with an evacuation order in 2016. The one-month prevalence of anxiety symptoms [32], PTSD [24], and depressive symptoms [24] were estimated at 19.8%, 12.8%, 14.8%, respectively, six months after the wildfire [32].

As aforementioned, FMM experienced three major disasters in five years by May of 2021, including the wildfires in 2016, the flooding in 2020, and the COVID-19 pandemic in 2020. We hypothesize that the three disasters increased mental health problems. The accumulative effect of the disasters may have led to a higher prevalence of PTSD-like symptoms among FMM residents than the prevalence prior to before the flood. We aimed to examine the prevalence of PTSD among FMM residents one year after the flood, and the sociological and clinical risk factors of likely PTSD. The further purpose was to improve understanding of the significance of the recovery experience in mental health outcomes. This could lead to improved provision of support to those potentially most vulnerable to future mental health problems because of natural disasters such as flooding [16].

## 2. Materials and Methods

### 2.1. Study Setting and Design

Fort McMurray (FMM) is in the Regional Municipality of Wood Buffalo in Northern Alberta, Canada, with a diverse population of 111,687 as of the 2018 census [23]. Through a cross-sectional survey introduced from 24 April to 2 June 2021, an online questionnaire was distributed randomly via email to residents of FMM, Canada, via REDCap [33]. Government, school, occupation and community were used as platforms to collect data. Sociodemographic, clinical, flooding-related information (e.g., Did you lose property because of the floods in Fort McMurray? Did you live in the same house you lived in before the floods? etc.), and the level of support received from different jurisdictions (e.g., support received from family; support received from the insurer, etc.) were collected. All residents of Fort McMurray who received the online survey link could participate in this study. There were no exclusion criteria based on clinical or demographic criteria. Participants were provided with information about the survey, and informed consent was implied by completing the survey questionnaires. Study approval was granted by the University of Alberta Research and Ethics Committee (Pro00066054).

### 2.2. Sample Size Estimation

With a population of 111,687 as of the 2018 census [23], a 95% confidence interval, and a ±3% margin of error, the sample size needed for prevalence rate estimates for PTSD will be 1058.

### 2.3. Outcome Measure

The PTSD checklist civilian version (PCL-C) [34] was used to assess the presence or absence of likely PTSD in respondents randomly selected from the natural settings in FMM. Many studies indicated that the PCL-C demonstrated good internal consistency and retest reliability. Compared with alternative measures of PTSD, the PCL-C showed favorable patterns of convergent and discriminant validity. Therefore, it is suggested as a psychometrically valid tool for screening and assessing the severity of PTSD in clinical practice and research [35,36,37,38]. There were, in total, 17 questions; the options varied from ‘significantly agree’ to ‘do not agree at all’. Patients with a PCL-C score of 44 or more were deemed to have likely PTSD [26,39,40].

### 2.4. Statistical Analysis

Data were analyzed using SPSS Version 25 [41]. Descriptive statistics were provided for demographic, clinical, and other variables against age. Chi-squared analyses were run to examine all the variables concerning the likely PTSD categorical variable. Logistic regression analysis was employed to identify significant risk factors of probable PTSD. We reported the responses without imputation of the missing data. Variables with a statistically significant relationship (*p* ≤ 0.05) or approaching significance (0.05 < *p* ≤ 0.1) to a likelihood of PTSD on univariate analysis were included in a logistic regression analysis. Correlational analysis was performed to determine any strong intercorrelations (Spearman’s correlation coefficient of 0.7 to 1.0 or −0.7 to −1.0) among predictor variables before applying logistic regression. Odds ratios from the binary logistic regression analysis were examined to determine the association between each variable in the model and the likelihood of respondents presenting with likely PTSD, controlling for the other variables.

## 3. Results

Among all the 249 participants who clicked on the survey link, 186 were returned with all key self-assessment questionnaires completed, giving a response rate of 74.7%. Descriptive demographic, clinical and flood-related feedback was collected from the whole sample of participants (N = 186), with age as a key variable (Table 1). Overall, the mean age of our respondents was 42 years; the majority of the sample were female 85.5% (159); 71% (132) of the respondents reported that they were in a relationship; 94.1% (175) were employed; 48.4% (90) reported having no mental health diagnosis, while 64.5% (120) reported not receiving psychotropic medications; 38.7% (72) reported receiving mental health counseling, and 52.7% (98) reported that they would like to receive mental health counseling; 65 respondents reported likely PTSD symptoms, yielding a prevalence of PTSD in our population (39.6%; 95% confidence interval: 32.1–47.6).

Regarding the flooding-related and received support information as mentioned above, 94.6% (176) of the respondents resided at FMM during the 2020 flood; 17.6% (31) were living in the flooded areas; respondents reported receiving a some-to-high level of support from family were 59.9% (100); from the Red Cross were 11.1% (19); from the Government of Alberta were 10.5% (18); from the insurer were 5.8% (10). More detailed characteristics of respondents are presented in Table 1.

### 3.1. Univariate Analysis

Associations between demographic, clinical antecedents, flooding-related experiences, and the likely PTSD were illustrated with the results of the univariate analysis in Table 2. We entered 41 predictor variables into the Chi-squared or Fisher exact test and found 14 predictor variables were significantly related to likely PTSD. Respondents who were not employed (100.0% (8)) were more likely to present with a likely PTSD compared to those who were employed (36.5% (57)). For the clinical characteristics, respondents with a history of depression (67.3% (35)), an anxiety disorder (59.4% (41)), or who had a history of mental health diagnosis before the flood (54.8% (46)) were more likely to present with likely PTSD compared to respondents with no history of depression (26.8% (30)), an anxiety disorder (25.3% (24)) or no mental health diagnosis history (23.8% (19)), respectively. Similarly, respondents who had a history of mental health counseling in the past year (62.3% (38)) and who would like to have mental health counseling (59.3% (51)) were more likely to show PTSD symptoms than those who did not have a history of mental health counseling (26.2% (27)) and those who would not like to receive mental health counseling (17.9% (14)). Furthermore, respondents who were on antidepressants (56.9% (29)), benzodiazepines (100.0% (4)), sleeping tablets (76.5% (13)), or who were on any medication for mental health concerns before the flood (53.4% (31)) were more likely to develop PTSD compared to respondents who were not on antidepressants (31.9% (36)), benzodiazepines (38.1% (61)), sleeping tablets (35.4% (52)) or no psychotropic medication (46.6% (27)), respectively.

Considering the disaster-related information, respondents who lived in the flooded area during the 2020 floods (58.6% (17)), who reported that their home suffered substantial damage because of the floods (88.9% (8)), and those who suffered loss of property in the floods (65.0% (13)) were more likely to present with probable PTSD compared to those who did not live in the flooding area during the 2020 floods (35.7% (46)), who did not suffer substantial damage because of the floods (36.8% (57)), and who suffered no loss of property in the floods (36.1% (52)). Finally, respondents who reported that they received no support from family (51.6% (32)) were more likely to present with PTSD compared to respondents who reported that they received some-to-high level of support from family (33.3% (32)), respectively. In addition, we found two variables approaching significance. Respondents who reported that their home suffered slight damage because of the floods (80.0% (4)) were more likely to report PTSD symptoms than those who reported that their home did not suffer any damage because of the flood (38.4% (61)). Respondents who reported receiving no support from their insurer (64.7% (11)) were more likely to present with probable PTSD compared to those who reported that they received a some-to-high level of support from the insurer (50.0% (5)).

### 3.2. Logistic Regression

Table 3 illustrates the multivariate logistic regression model that predicts likely PTSD among study participants. In total, 16 predictors were found with significant or near-significant *p* values (*p* ≤ 0.1) (Table 2). Never received a mental health diagnosis from a health professional, history of antidepressant medications, and suffered no loss of property in the floods were dropped from the regression model, as they were highly positively correlated with other variables (rs > 0.7). Therefore, we ended up entering 13 variables into the logistic regression model to predict the likelihood of PTSD.

The model was statistically significant; Χ2 (df = 14; n = 152) = 84.98, *p* ≤ 0.001, suggesting that the model could distinguish between respondents who did or did not exhibit likely PTSD one year after the 2020 floods in FMM. The model explained 42.8% (Cox and Snell R2) and 57.8% (Nagelkerke R2) of the variance in indicating the likelihood that respondents will present with PTSD and correctly classified 80.3% of cases.

Among all the variables, only three of the predictors, having received depression diagnosis from a health professional, would like to receive mental health counseling and received limited or no support from family exhibited significant prediction of likely PTSD in the model. Respondents who reported that they received a depression diagnosis from a health professional were more than five times more likely to report likely PTSD symptoms because of floods, compared to those who did not (OR = 5.71; 95% CI: 1.68–19.36) while controlling for the other variables. Similarly, respondents who reported they would like to receive mental health counseling were almost three times more likely to report likely PTSD symptoms after floods, compared to those who were not willing to receive mental health counseling (OR = 2.87; 95% CI: 1.02–8.05), after controlling for other predictors. Moreover, respondents who reported that they received limited or no support from their family were almost three times more likely to express likely PTSD symptoms than those who reported receiving some-to-high levels of support from their families (OR = 2.87; 95% CI: 1.06–7.74), after controlling for other model variables. The largest contribution to the model was provided by the received depression diagnosis (Wald = 7.82).

## 4. Discussion

Our research evaluated the prevalence and the potential risk factors of likely PTSD, one year after the FMM flooding in 2020 among residents of FMM who participated in the study. In this study, the prevalence of likely PTSD among respondents was 39.6%. Unlike our stated hypothesis, this outcome is consistent with findings from previous studies which estimate the prevalence of PTSD to be 30–40% among direct victims, 10–20% among rescue workers, and 5–10% in the general population of FMM [9,34,42,43]. What aligned with our prediction is that the prevalence of likely PTSD in our study (one year after the flood) was much higher when compared with the prevalence of 12.8% and 13.6% in the FMM residents 6 months and 18 months after a 2016 wildfire [24,41]. This might be explained, as our study was conducted among a population that has experienced multiple traumatic events over the past 5 years (2016’s wildfire, 2020’s COVID and Flood). Thus, there is a potential additive effect that may explain why the rate of PTSD was higher than with the previous studies on wildfires. The time of sampling may be another significant factor, where the current study was carried out during the continuous COVID-19 pandemic when there was still so much uncertainty, while the wildfire surveys were conducted at 6 and 18 months after the wildfires when there was no pandemic. Future studies that evaluate multiple traumas on the population would be expected to clarify this.

None of the demographic characteristics showed an association with likely PTSD after flooding. Although compared to males and older age groups, females and the younger age group expressed a higher prevalence of PTSD symptoms, the difference was slight and not significant. This conflicts with previous studies, where females and younger age groups were indicated to be more likely to express mental health problems, including likely PTSD symptoms, compared to males and older age groups [44,45,46,47,48]. Since our study sample size was relatively small, it may not have elicited that difference as reported in other studies.

Among clinical predictors, prior depression history was a significant risk factor for the likelihood of PTSD. The majority of respondents who had a depression history (67.3%) reported having likely PTSD symptoms after the flood. This finding is highly consistent with research on the main effects of prior mental health disorders on PTSD [3,45,49,50,51,52]. Much research has determined a relationship between depression and PTSD among those who have experienced trauma, including natural disasters, childhood maltreatment, serious accident, or terrorist act [53,54,55]. Worthington’s research had identified past borderline personality disorder diagnosis and past depression as two of the most significant prediction for the onset of PTSD after the disaster [45]. In addition, a study with 1820 respondents who supported the mission in Afghanistan [51] showed that the prevalence of PTSD was highest among those with a pre-deployment history of depression [51]. This might suggest that past psychiatric diagnoses such as depression may successfully predict future mental health disorders [51]. Likewise, in China, a cross-sectional study aimed to investigate people’s mental health conditions during the COVID-19 pandemic reported that more than one-third of the study participants who met diagnostic criteria for PTSD had a psychiatric diagnosis history [49,56]. A similar result can also be found in a long-term study of the SARS-CoV-1 pandemic [50].

The connection between PTSD and the prior depression history could be interpreted in a number of ways [57]. One possibility is that people who have depression show a higher possibility to encounter traumatic experiences than those without depression, which, in turn, may improve the possibilities of the onset of PTSD [57]. The other explanation is about the gene. Family history is a significant predictor for the development of depression. Therefore, it makes sense that some specific genetic factors might play a role in the appearance of both depression and PTSD [5].

Family support was found to have a sign of significantly associated valence with likely PTSD. In our study, respondents who reported receiving limited or no support from their family were more likely to show PTSD symptoms than their counterparts who received some-to-high level of family support. Consistent with our finding, family supports have long been regarded as protective solid or resource factors in the aftermath of trauma. Sufficient social support, primarily coming from family, before the disaster, can reduce the adverse psychological effects of natural disaster exposure and improve coping and resilience [31,58,59,60,61]. Conversely, lack of social support in close family members friends before a traumatic event has been associated with the development of PTSD in numerous populations following the disaster [62,63,64]. Previous studies conducted in FMM after the devastating wildfire in 2016 indicated that having only limited or no support from friends and family is one of the significant risk factors for likely PTSD among the residents of FMM post-disaster [9,43,65]. This finding is consistent with the longitudinal study about crisis support following the MS Herald of Free Enterprise disaster [9,66]; the study aimed at exploring the coping strategies for technological disasters [67], the research about psychiatric disorders among victims of courthouse shootings [68], and the exploration about psychiatric disorders among survivors of the Oklahoma City Bombing [69].

The willingness to receive mental health counseling after the disaster was another predictor in our study. In published studies, there is not much data addressing the risk factors associated with post-disaster counselling. According to some research, counseling may not relieve symptoms of psychiatric disorders such as PTSD after disaster events [70]. For example, according to the 2002 Cochran review, post-trauma debriefing may offer no PTSD preventative benefits and might potentially be harmful [70,71]. Therefore, our finding may offer explanation as to why participants who experienced PTSD (low resilience) preferred to seek counseling assistance after experiencing intensive distress and mental health problems. In the future, studies with more concrete output on events pre and post disasters are required to indicate any negative relationship between seeking counseling and mental health outcomes.

### Limitations of the Study

Our study has some limitations that need to be considered when interpreting the findings. Firstly, this study was conducted during the COVID-19 pandemic; thus, the panic might have contributed to the high PTSD symptoms in study participants. Moreover, the survey link was sent by the community providers; thus, there was no way of knowing the actual number of people who received the survey. Therefore, the response rate in our study may be overestimated, as it is based only on the number of people who clicked the survey link instead of the number of people who received the survey. Besides, our sample size of 186 was much less than the sample size estimates that we projected. As such, the margin error of our prevalence estimates was ±7% rather than the ±3%, which we projected initially. Due to the unbalanced groups in our data, some variables were dropped from the analysis which might have confounded the results of this study. In addition, there was an over-representation of a history of mental health conditions in respondents in this study. In addition, all the symptom-related responses were self-reported and were not based on clinical assessment, which may undermine the validity of the study findings. Moreover, we could not collect data from all categories of FMM inhabitants, such as the workers in remote camps. Despite these limitations, our study may be considered as an accurate reflection of respondents from the sites sampled and, thus, useful for policy purposes.

## 5. Conclusions

This study has evaluated the prevalence of PTSD among the residents of FMM one year after the devastating flood and has determined the demographic, clinical, disaster-related, and other risk variables of likely PTSD in the respondents. Our findings indicated that one year after the FMM flood in 2020, 39.6% of the respondents (40.8% females and 31.8% males) keep suffering from probable PTSD. Consistent with previous studies, perceived family and social support were one of the solid protective factors. It would be useful for future studies and policy practitioners to investigate more closely the interaction between emotional support that survivors can receive from family, friends and material support offered by public institutions or charities in the context of mental wellness and recovery. For example, the lessons learned from the 2016 FMM wildfires were successfully applied to the 2018 wildfire in British Columbia. These were mainly about cooperation and communication among various relief organizations and more efforts to build community partnerships, which could improve mental health support during the recovery process for the affected communities [32]. In addition, receiving a mental health depression diagnosis from a health professional before the disaster and wanting to receive mental health counseling after the disaster were significant risk predictors for suffering from a likely PTSD. This information might have significant reference value for policymakers when crafting crisis response programs at a population level to alleviate the effects of natural disasters on the public’s mental health. More in-depth studies in the future are required to explore the possible association between counseling and probable PTSD symptoms. On top of that, preparedness plans for future disasters might need to provide broad mental health resilience education to the residents in a high-risk area. A variety of innovative programs or mobile health options [24,32,65] that can provide cost-effective, self-directed, and location-independent interventions that can enhance accessibility and continuity of care in situations where resources are scare should be introduced to high-risk groups, in particular, to help policymakers effectively process the post-disaster mental help in the future.

## Figures and Tables

**Table 1:** Demographic information, clinical characteristics and support received by respondents.

Variables	≤30 y n (%)	31–40 y n (%)	41–50 y n (%)	>50 y n (%)	Total n (%)
**Gender**					
Male	4 (12.9)	5 (8.8%)	11 (20.0)	7 (16.3)	27 (14.5)
Female	27 (87.1)	52 (91.2)	44 (80.0)	36 (83.7)	159 (85.5)
**Employment status**					
Employed	25 (80.6)	57 (100.0)	52 (94.5)	41 (95.3)	175 (94.1)
Unemployed	6 (19.4)	0 (0.0)	3 (5.5)	2 (4.7)	11 (5.9)
**Relationship status**					
In a relationship	16 (51.6)	46 (80.7)	39 (70.9)	31 (72.1)	132 (71.0)
Not in a relationship	15 (48.4)	11 (19.3)	16 (29.1)	12 (27.9)	54 (29.0)
**Housing status**					
Own home	20 (64.5)	41 (71.9)	42 (76.4)	38 (88.4)	141 (75.8)
Renting	11 (35.5)	16 (28.1)	13 (23.6)	5 (11.6)	45 (24.2)
**History of mental health diagnosis from a health professional**					
Depressive disorder	9 (29.0)	18 (31.6)	20 (36.4)	11 (25.6)	58 (31.2)
Bipolar disorder	0 (0.0)	2 (3.5)	3 (5.5)	1 (2.3)	6 (3.2)
Anxiety disorder	11 (35.5)	28 (49.1)	27 (49.1)	12 (27.9)	78 (41.9)
Alcohol abuse	1 (3.2)	0 (0.0)	1 (1.8)	1 (2.3)	3 (1.6)
Drug abuse	0 (0.0)	0 (0.0)	1 (1.8)	1 (2.3)	2 (1.1)
Personal disorder	0 (0.0)	1 (1.8)	1 (1.8)	0 (0.0)	2 (1.1)
Others	3 (9.7)	7 (12.3)	4 (7.3)	3 (7.0)	17 (9.1)
No mental health diagnosis	16 (51.6)	23 (40.4)	26 (47.3)	25 (58.1)	90 (48.4)
**History of psychotropic medications**					
Antidepressants	10 (32.3)	17 (29.8)	22 (40.0)	10 (23.3)	59 (31.7)
Antipsychotics	1 (3.2)	1 (1.8)	2 (3.6)	0 (0.0)	4 (2.2)
Benzodiazepines	2 (6.5)	1 (1.8)	0 (0.0)	1 (2.3)	4 (2.2)
Mood stabilizers	2 (6.5)	3 (5.3)	4 (7.3)	3 (7.0)	12 (6.5)
Sleeping tablets	5 (16.1)	4 (7.0)	7 (12.7)	5 (11.6)	21 (11.3)
Other	0 (0.0)	1 (1.8)	1 (1.8)	1 (2.3)	3 (1.6)
Not on psychotropic medication	20 (64.5)	37 (64.9)	33 (60.0)	30 (69.8)	120 (64.5)
**Respondents who received MH counseling in the past year**	18 (58.1)	24 (42.1)	19 (34.5)	11 (25.6)	72 (38.7)
**Respondents who would like to receive MH counseling**	22 (71.0)	33 (57.9)	29 (52.7)	14 (32.6)	98 (52.7)
**Respondents who resided at Fort McMurray during the 2020 flood**	28 (90.3)	53 (93.0)	52 (94.5)	43 (100.0)	176 (94.6)
**Respondents who resided in the flooding areas**	3 (10.7)	8 (15.1)	8 (15.4)	12 (27.9)	31 (17.6)
**Respondents who witnessed the flooding of homes or structures in For McMurray**	18 (69.2)	42 (77.8)	37 (72.5)	34 (79.1)	131 (75.3)
**Respondents who have been fearful for their own lives, or the lives of their close friends or family members**	6 (23.1)	19 (35.2)	16 (31.4)	10 (23.3)	51 (29.3)
**During the 2020 Fort McMurray flooding, how frequent did you watch TV images about the devastation caused by the flood?**					
Daily	11 (42.3)	35 (64.8)	40 (78.4)	30 (69.8)	116 (66.7)
<Daily	12 (46.2)	8 (14.8)	8 (15.7)	9 (20.9)	37 (21.3)
I did not watch TV images about the devastation	3 (11.5)	11 (20.4)	3 (5.9)	4 (9.3)	21 (12.1)
**During the 2020 Fort McMurray flooding, how frequent did you read newspaper and internet articles related to devastation caused by the flood?**					
Daily	16 (61.5)	46 (85.2)	39 (78.0)	31 (72.1)	132 (76.3)
<Daily	9 (34.6)	7 (13.0)	10 (20.0)	9 (20.9)	35 (20.2)
I did not read newspaper or articles about the devastation	1 (3.8)	1 (1.9)	1 (2.0)	3 (7.0)	6(3.5)
**Did you lose property because of the floods in Fort McMurray?**					
Home suffered substantial damage	0 (0.0)	2 (3.5)	2 (3.6)	5 (11.6)	9 (4.8)
Home suffered slight damage	0 (0.0)	2 (3.5)	2 (3.6)	1 (2.3)	5 (2.7)
Car was destroyed by the floods	0 (0.0)	0 (0.0)	1 (1.8)	3 (7.0)	4 (2.2)
Business was destroyed by the floods	0 (0.0)	3 (5.3)	2 (3.6)	1 (2.3)	6 (3.2)
Suffered no loss of property in the floods	31 (100.0)	50 (87.7)	49 (89.1)	35 (81.4)	165 (88.7)
**Do you live in the same house you lived in before the floods?**					
Same house	21 (80.8)	47 (88.7)	40 (78.4)	39 (90.7)	147 (85.0)
Different house—previous home not destroyed by the fire	5 (19.2)	5 (9.4)	8 (15.7)	4 (9.3)	22 (12.7)
Different home—previous home destroyed by the flood	0 (0.0)	1 (1.9)	3 (5.9)	0 (0.0)	4 (2.3)
**Support received from family**					
Some-to-high level of support	17 (65.4)	34 (65.4)	25 (52.1)	24 (58.5)	100 (59.9)
Limited or no support	9 (34.6)	18 (34.6)	23 (47.9)	17 (41.5)	67 (40.1)
**Support received from Red Cross**					
Some-to-high level of support	3 (11.5)	3 (5.7)	6 (12.2)	7 (16.3)	19 (11.1)
Limited or no support	1 (3.8)	3 (5.7)	7 (14.3)	6 (14.0)	17 (9.9)
NA (not impacted by the flood)	22 (84.6)	47 (88.7)	36 (73.5)	30 (69.8)	135 (78.9)
**Support received from the Government of Alberta**					
Some-to-high level of support	4 (15.4)	2 (3.8)	7 (14.3)	5 (11.6)	18 (10.5)
Limited or no support	0 (0.0)	4 (7.5)	6 (12.2)	8 (18.6)	18 (10.5)
NA (not impacted by the flood)	24 (92.3)	47 (88.7)	36 (73.5)	30 (69.8)	135 (78.9)
**Support received from Insurer**					
Some-to-high level of support	2 (7.7)	2 (3.8)	2 (4.1)	4 (9.3)	10 (5.8)
Limited or no support	0 (0.0)	4 (7.5)	7 (14.3)	7 (16.3)	18 (10.5)
NA (not impacted by the flood)	24 (92.3)	47 (88.7)	40 (81.6)	32 (74.4)	143 (83.6

MH: Mental health.

**Table 2:** Chi-squared test of association between demographic, clinical antecedents & likely PTSD.

Variables	Unlikely PTSD	Likely PTSD	Chi-Square/Fisher Exact	*p*-Value	Phi Value
**Demographic Characteristics**					
**Gender**					
Male	15 (68.2)	7 (31.8)			
Female	84 (59.2)	58 (40.8)	0.65	0.49	0.06
**Age categories**					
≤40 y	44 (58.7)	31 (41.3)			
>40 y	55 (61.8)	34 (38.2)	0.17	0.75	−0.03
**Employment status**					
Employed	99 (63.5)	57 (36.5)			
Unemployed	0 (0.0)	8 (100)	12.81	<0.001 *	0.28
**Employment place**					
School boards	54 (72.0)	21 (28.0)			
Healthcare industry	5 (62.5)	3 (37.5)			
Keyano College	12 (60.0)	8 (40.0)			
Oil Sands industry	5 (41.7)	7 (58.3)			
Municipal or government agency	8 (66.7)	4 (33.3)			
Other	14 (50.0)	14 (50.0)	7.14	0.21	0.22
Relationship					
In a relationship	76 (63.3)	44 (36.7)			
Not in a relationship	23 (52.3)	21 (47.7)	1.65	0.21	0.10
**Clinical Characteristics**					
**History of Depression diagnosis from a health professional?**					
No	82 (73.2)	30 (26.8)			
Yes	17 (32.7)	35 (67.3)	24.37	<0.001 *	0.39
**History of Bipolar diagnoses from a health professional?**					
No	96 (60.4)	63 (39.6)			
Yes	3 (60.0)	2 (40.0)	0.00	0.99	−0.00
**History of Anxiety diagnosis from a health professional?**					
No	71 (74.7)	24 (25.3)			
Yes	28 (40.6)	41 (59.4)	19.49	<0.001 *	0.35
**History of Alcohol Abuse diagnosis from a health professional?**					
No	97 (60.2)	64 (39.8)			
Yes	2 (66.7)	1 (33.3)	0.05	0.99	0.02
**History of Drug Abuse diagnosis from a health professional?**					
No	98 (60.5)	64 (39.5)			
Yes	1 (50.0)	1 (50.0)	0.09	0.99	−0.02
**History of Personality Disorder diagnosis from a health professional?**					
No	98 (60.1)	65 (39.9)			
Yes	1 (100.0)	0 (0.0)	0.66	0.99	0.06
**History of Other diagnosis from a health professional?**					
No	90 (60.4)	59 (39.6)			
Yes	9 (60.0)	6 (40.0)	0.00	0.99	−0.00
**Never received a mental health diagnosis from a health professional**					
No	61 (76.3)	19 (23.8)			
Yes, received MH Dx	38 (45.2)	46 (54.8)	16.47	<0.001 *	−0.32
**History of mental health counseling in the past year**					
No	76 (73.8)	27 (26.2)			
Yes	23 (37.7)	38 (62.3)	20.84	<0.001 *	0.36
**Would you like to receive mental health counseling?**					
No	64 (82.1)	14 (17.9)			
Yes	35 (40.7)	51 (59.3)	29.23	<0.001 *	0.42
**History of Antidepressant medications**					
No	77 (68.1)	36 (31.9)			
Yes	22 (43.1)	29 (56.9)	9.18	<0.01 *	−0.24
**History of Antipsychotic medications**					
No	98 (60.5)	64 (39.5)			
Yes	1 (50.0)	1 (50.0)	0.09	0.99	0.02
**History of Benzodiazepine medications**					
No	99 (61.9)	61 (38.1)			
Yes	0 (0.0)	4 (100.0)	6.25	0.02 *	0.20
**History of Mood Stabilizer medications**					
No	95 (61.3)	60 (38.7)			
Yes	4 (44.4)	5 (55.6)	1.01	0.49	0.08
**History of Sleeping Tablets**					
No	95 (64.6)	52 (35.4)			
Yes	4 (23.5)	13 (76.5)	10.76	≤0.001 *	0.26
**History of Other mental health medications not listed**					
No	96 (59.6)	65 (40.4)			
Yes	3 (100.0)	0 (0.0)	2.00	0.28	−0.11
**Not on any medication for mental health concerns**					
No	72 (67.9)	34 (32.1)			
Yes, on MH Mx	27 (46.6)	31 (53.4)	7.16	0.01 *	−0.21
**Fort McMurray Flood Related Characteristics**					
**Did you reside at Fort McMurray during the 2020 flood?**					
No	4 (66.7)	2 (33.3)			
Yes	95 (60.1)	63 (39.9)	0.10	0.99	0.03
**Areas of residence during the 2020 flood**					
Not in the flooding area	83 (64.3)	46 (35.7)			
In the flooding area	12 (41.4)	17 (58.6)	5.20	0.04 *	0.18
**Housing status prior flood**					
Own home	80 (63.5)	46 (36.5)			
Renting	19 (50.0)	19 (50.0)	2.22	0.19	0.12
**Housing status now**					
Own home	78 (60.0)	52 (40.0)			
Renting	21 (61.8)	13 (38.2)	0.04	0.99	−0.02
**Where did you live just prior to the 2020 flooding?**					
In Fort McMurray	95 (60.1)	63 (39.9)			
Other	4 (66.7)	2 (33.3)	0.10	0.99	−0.03
**Did you witness the flooding of any homes or structures in Fort McMurray?**					
No	25 (62.5)	15 (37.5)			
Yes	74 (59.7)	50 (40.3)	0.10	0.85	0.03
**During the flooding, were you fearful for your life or the lives of your friends or family?**					
No	74 (64.3)	41 (35.7)			
Yes	25 (51.0)	24 (49.0)	2.55	0.12	0.13
**During the 2020 Fort McMurray flooding, how frequently did you watch television images about the devastation caused by the flood?**					
Daily	61 (56.0)	48 (44.0)			
<Daily	25 (71.4)	10 (28.6)			
I did not watch the TV images of the devastation.	13 (65.0)	7 (35.0)	2.85	0.24	0.13
**During the 2020 Fort McMurray flooding, how frequently did you read newspaper and internet articles related to the devastation caused by the flooding?**					
Daily	73 (57.9)	53 (42.1)			
<Daily	22 (66.7)	11 (33.3)			
I did not read newspaper and internet articles related to the devastation.	4 (80.0)	1 (20.0)	1.66	0.40	0.10
**Respondents who report their home suffered substantial damage because of the floods in Fort McMurray**					
Unchecked	98 (63.2)	57 (36.8)			
Checked	1 (11.1)	8 (88.9)	9.66	<0.01 *	0.24
**Respondents who report their home suffered slightly damage because of the floods in Fort McMurray**					
Unchecked	98 (61.6)	61 (38.4)			
Checked	1 (20.0)	4 (80.0)	3.51	0.08	0.15
**Respondents report their car was completely destroyed by the floods in Fort McMurray**					
Unchecked	96 (60.0)	64 (40.0)			
Checked	3 (75.0)	1 (25.0)	0.37	0.65	−0.05
**Respondents report their business was completely destroyed by the floods in Fort McMurray**					
Unchecked	95 (59.7)	64 (40.3)			
Checked	4 (80.0)	1 (20.0)	0.83	0.65	−0.07
**Respondents report they suffered no loss of property in the floods**					
No, did not lose	92 (63.9)	52 (36.1)			
Yes, lose	7 (35.0)	13 (65.0)	6.12	0.02 *	0.19
**Do you live in the same house you lived in before the floods?**					
Yes	86 (61.9)	53 (38.1)			
No, I live in a different house even though my previous home was not destroyed by the flood.	11 (55.0)	9 (45.0)			
No, I live in a different house because my previous home was destroyed by the flood.	1 (25.0)	3 (75.0)	2.45	0.35	0.12
**Support received from Family**					
Some-to-high level of support	64 (66.7)	32 (33.3)			
Limited or not support	30 (48.4)	32 (51.6)	5.22	0.03 *	0.18
**Support received from Red Cross**					
Some-to-high level of support	8 (47.1)	9 (52.9)			
Limited or not support	8 (47.1)	9 (52.9)	2.94	0.23	0.14
**Support received from Government of Alberta**					
Some-to-high level of support	7 (41.2)	10 (58.8)			
Limited or not support	9 (52.9)	8 (47.1)	3.43	0.18	0.15
**Support received from Insurer**					
Some-to-high level of support					
Limited or not support	5 (50.0)	5 (50.0)			
NA (not impacted by the flood)	6 (35.3)	11 (64.7)	5.5	0.06	0.18

* *p* ≤ 0.05.

**Table 3:** Logistic regression predicting likelihood of residents presenting with PTSD.

Predictor	B	S.E.	Wald	df	*p* Value	Odds Ratio	95% CI for Odds Ratio	
							Upper Lower	
**Currently employed**	−22.24	12,156.65	0.00	1	0.99	0.00	0.00	.
**Having received Depression diagnosis from a health professional**	1.74	0.62	7.82	1	0.01 *	5.71	1.68	19.36
**Having received Anxiety diagnosis from a health professional**	0.90	0.59	2.34	1	0.13	2.47	0.78	7.86
**Having received mental health counseling in the past year**	0.92	0.58	2.54	1	0.11	2.51	0.81	7.78
**Would like to receive mental health counseling**	1.06	0.53	4.02	1	0.05 *	2.87	1.02	8.05
**On Benzodiazepines for a mental health concern**	−20.16	15,770.89	0.00	1	0.99	0.00	0.00	.
**On Sleeping Tablets**	−0.99	0.97	1.05	1	0.31	0.37	0.06	2.48
**Not on any medication for a mental health concern**	1.17	0.70	2.78	1	0.09	3.22	0.81	13.00
**Live in the area of flooding during the 2020 flood**	0.11	0.80	0.02	1	0.89	1.11	0.23	5.36
**Home suffered substantial damage as a result of the floods in Fort McMurray**	3.42	2.19	2.44	1	0.12	30.69	0.42	2250.16
**Home suffered slight damage as a result of the floods in Fort McMurray.**	2.18	1.60	1.84	1	0.18	8.82	0.38	204.61
**Received limited or no support from family**	1.05	0.51	4.33	1	0.04 *	2.87	1.06	7.74
**Received some-to-high level of support from insurer**			1.65	2	0.44			
**Received limited or no support from insurer**	2.03	1.70	1.43	1	0.23	7.61	0.27	213.07
**Not impact by the flood**	1.35	1.67	0.65	1	0.42	3.84	0.15	101.93
**Constant**	38.03	19,912.434	0.000	1	0.99	3.30 × 10^16^		

* Significance at *p* ≤ 0.05 CI: confidence interval. S.E.: standard error. df: degree of freedom.

## Data Availability

Data is available upon reasonable request to the submitting author.
